# Long-term risk of ischemic heart disease after adjuvant radiotherapy in breast cancer: results from a large population-based cohort

**DOI:** 10.1186/s13058-020-1249-2

**Published:** 2020-01-22

**Authors:** Anna-Karin Wennstig, Charlotta Wadsten, Hans Garmo, Irma Fredriksson, Carl Blomqvist, Lars Holmberg, Greger Nilsson, Malin Sund

**Affiliations:** 1 Department of Surgical and Perioperative Sciences, Umeå University, Umeå, Sweden; 2 Department of Oncology, Sundsvall Hospital, Sundsvall, Sweden; 3 Department of Surgery, Sundsvall Hospital, Sundsvall, Sweden; 4 Regional Cancer Center, Uppsala University/Uppsala University Hospital, Uppsala, Sweden; 5 Translational Oncology & Urology Research (TOUR), School of Cancer and Pharmaceutical Sciences, King’s College London, London, UK; 6 Department of Breast-and Endocrine Surgery, Karolinska University Hospital, Stockholm, Sweden; 7 Department of Molecular Medicine and Surgery, Karolinska Institutet, Stockholm, Sweden; 8 Department of Oncology, Örebro University, University Hospital, Örebro, Sweden; 9 Department of Surgical Sciences, Uppsala University, Uppsala, Sweden; 10 Department of Immunology, Genetics and Pathology, Section of Experimental and Clinical Oncology, Uppsala University, University Hospital, Uppsala, Sweden; 11 Department of Oncology, Gävle Hospital, Gävle, Sweden; 12 Department of Oncology, Visby Hospital, Visby, Sweden

**Keywords:** Breast cancer, Radiotherapy, Survivorship, Ischemic heart disease, Long-term side effects

## Abstract

**Background:**

Adjuvant radiotherapy (RT) for breast cancer (BC) has been associated with an increased risk of ischemic heart disease (IHD). We examined the incidence of IHD in a large population-based cohort of women with BC.

**Methods:**

The Breast Cancer DataBase Sweden (BCBaSe) includes all women diagnosed with BC from 1992 to 2012 (*n* = 60,217) and age-matched women without a history of BC (*n* = 300,791) in three Swedish health care regions. Information on comorbidity, educational level, and incidence of IHD was obtained through linkage with population-based registries. The risk of IHD was estimated by Cox proportional hazard regression analyses and cumulative incidence by the Kaplan-Meier method.

**Results:**

Women with BC had a lower risk of IHD compared to women without BC with a hazard ratio (HR) of 0.91 (95% CI 0.88–0.95). When women with left-sided BC were compared to right-sided BC, an increased HR for IHD of 1.09 (95% CI 1.01–1.17) was seen. In women receiving RT, a HR of 1.18 (95% CI 1.06–1.31) was seen in left-sided compared to right-sided BC, and the HRs increased with more extensive lymph node involvement and with the addition of systemic therapy. The cumulative IHD incidence was increased in women receiving left-sided RT compared to right-sided RT, starting from the first years after RT and sustained with longer follow-up.

**Conclusions:**

Women given RT for left-sided BC during 1992 to 2012 had an increased risk of IHD compared to women treated for right-sided BC. These women were treated in the era of three-dimensional conformal RT (3DCRT), and the results emphasize the importance of further developing and implementing RT techniques that lower the cardiac doses, without compromising the beneficial effects of RT.

## Background

Radiotherapy (RT) is associated with ischemic heart disease (IHD) due to incidental cardiac radiation exposure, and the IHD risk increases linearly with the radiation dose to the heart and coronary arteries without an apparent threshold dose [1–3]. Women irradiated for left-sided breast cancer (BC) receive substantially higher doses to the heart compared to women irradiated for right-sided BC, and several trials show an increased IHD risk after RT in left-sided BC [1, 4, 5]. A higher incidence of stenosis in the left anterior descending artery (LAD) has been reported after RT of left-sided compared to right-sided BC [5–8]. A large population-based study in women with BC treated from 1976 to 2006 showed an increase in acute myocardial infarction, angina pectoris, pericarditis, and valvular heart disease after left-sided RT compared to right-sided RT, with an enhanced risk in women diagnosed with IHD prior to BC [1]. The development of new RT techniques has decreased radiation doses to the heart. Even so, doses to the anterior part of the heart, especially the LAD, may still be high [9–11].

Many women with BC receive endocrine therapy and chemotherapy in addition to RT. Studies have shown a higher risk of cardiovascular disease in patients treated with aromatase inhibitors (AI) compared to tamoxifen, though this elevation in risk may be explained by a cardioprotective effect of tamoxifen [12, 13]. Cytotoxic agents, especially regimens including anthracyclines, are associated with an increased risk of congestive heart failure [14, 15]. Anthracyclines combined with RT have been associated with a higher risk of heart disease in left-sided BC compared to RT alone, suggesting an additive effect [16, 17]. The aim of the present study was to examine the risk of IHD in women with BC receiving RT and age-matched women without a history of BC in a large population-based cohort. The possible added risk from combining RT with endocrine therapy and chemotherapy was also studied.

## Methods

### Study population

Since the late 1970s, information on Swedish women with BC has been recorded in regional breast cancer registries. From 2008, all BC patients are registered in the National Quality Registry for Breast Cancer. For research purposes, the registries from three of Sweden’s six health care regions (Stockholm, Uppsala-Örebro, and the Northern region) were merged to form the Breast Cancer DataBase Sweden (BCBaSe) cohort. All new cases of invasive BC in women from 1992 to 2012 were included. For each BC case, five women without a BC diagnosis, born in the same year as the index case, were added to form a comparison cohort. Women of the comparison cohort were allowed to become BC cases if diagnosed with BC during the study period. In the present study, all patients with early BC were selected from the BCBaSe cohort, and patients with metastatic BC at diagnosis were excluded.

The study cohort was linked to the National Patient Register (NPR) and the Longitudinal Integration Database for Health Insurance and Labor Market Studies, using the unique personal identity numbers issued to all Swedish citizens. NPR is a validated registry containing records of all hospital discharges in Sweden since 1987 and also hospital-based outpatient care since 2001 [18]. The NPR contains information on the main diagnosis and up to eight secondary diagnoses. The Longitudinal Integration Database for Health Insurance and Labor Market Studies records data from the labor market and the educational and social sectors. This registry contains data on socioeconomic variables for all Swedish residents, such as marital status, income, place of employment, and the highest level of education [19].

Comorbidity was classified using the NPR according to the Charlson Comorbidity Index (CCI) in three comorbidity levels: CCI 0 (no comorbidity), CCI 1 (mild comorbidity), and CCI 2 (severe comorbidity) [20].

### Statistical methods

IHD was defined according to the International Classification of Disease (ICD) 9th edition codes 410–414 or ICD-10 codes I20–I25, which include angina pectoris, acute myocardial infarction, complications due to myocardial infarction, and chronic IHD. To estimate the risk of IHD, Cox proportional hazard regression analysis was performed, using age as a timescale. The risk of IHD was examined by comparing women with BC to women without BC and by comparing women receiving left-sided RT to right-sided RT. The analyses were stratified for the type of surgery, RT, endocrine therapy, chemotherapy, and treatment with trastuzumab. The RT was also stratified based on the pathological lymph node status recorded in the breast cancer registries. According to the Regional BC treatment guidelines during the study period, women with no lymph node metastases were likely to receive RT to the breast or chest wall alone, and women with more than four positive lymph nodes were likely to receive loco-regional RT. The Regional BC treatment guidelines concerning RT in women with one to three positive lymph nodes varied over time and between the different regions included. This group most likely includes both women that received RT to the breast or chest wall alone and women that received loco-regional RT. Women with bilateral BC and women with unknown BC laterality were excluded in all analyses regarding the risk of IHD by laterality. Analyses concerning trastuzumab included only women with BC diagnosed from the year 2005.

The analyses were adjusted for the number of previous IHD events, time since last IHD event, non-cardiac comorbidity (CCI with IHD removed), and educational level (low, < 10 years mandatory school; intermediate, 10–12 years high school; and high, university or college). Women with BC were followed from the date of BC diagnosis (i.e., date of inclusion) to the date of the first IHD event registered in the NPR after inclusion, death, migration, or at the end of follow-up (31 December 2013), whichever came first. For women without BC in the comparison cohort who later were diagnosed with BC, an additional end of follow-up was defined. Cumulative incidence of IHD was calculated by the Kaplan-Meier method separately for all women with BC; women with BC receiving left-sided RT, right-sided RT, or no RT; and women without BC. Analyses were performed using the statistical software R [21].

## Results

### Study population

The study population consisted of 60,217 women with BC and 300,791 women without a history of BC, shown in Table 1. There were 28,903 women diagnosed with right-sided BC and 30,840 with left-sided BC, while 474 women had bilateral BC or unknown BC laterality. The mean follow-up time was 8.1 years for women with BC and 9.0 years in women without BC. The mean age at inclusion was 62.6 years. Women with BC had a higher educational level compared to women without BC diagnosis; 27.6% of the women with BC had an education longer than 12 years compared to 24.4% of the women without BC. No major differences were seen between women with BC and women without BC concerning CCI or a history of previous IHD events. The vast majority of women in the study population had no reported comorbidity. Previous IHD events were reported in 3.9% of the women with BC and in 4.2% of the women without BC. Women with BC who received no RT were older at the time of BC diagnosis compared to women with BC who received RT, 69 years vs. 60 years, respectively; had more comorbidities according to CCI (89% vs. 99% had CCI = 0); and were more frequently diagnosed with IHD (3.5% vs. 1%) prior to BC diagnosis (data not shown in the table).

**Table 1:** Characteristics of the study population

	BC all, *n* (%)	BC right, *n* (%)	BC left, *n* (%)	Women without BC, *n* (%)
No. of women	60,217	28,903	30,840	300,791
FU time, yrs (SD)	8.1 (5.5)	8.1 (5.5)	8.1 (5.5)	9.0 (5.6)
Health care region				
Stockholm	25,606 (42.5)	12,448 (43.1)	13,158 (42.7)	127,937 (42.5)
Uppsala/Örebro	25,323 (42.1)	11,919 (41.2)	12,934 (41.9)	126,490 (42.1)
Northern region	9288 (15.4)	4536 (15.7)	4748 (15.4)	46,364 (15.4)
Year of inclusion				
1992–1997	14,149 (23.5)	6578 (22.8)	7103 (23.0)	70,666 (23.5)
1998–2002	14,285 (23.7)	6960 (24.1)	7320 (23.7)	71,362 (23.7)
2003–2007	14,960 (24.8)	7210 (24.9)	7749 (25.1)	74,740 (24.8)
2008–2012	16,823 (27.9)	8155 (28.2)	8668 (28.1)	84,023 (27.9)
Age at inclusion				
< 45 yrs	5770 (9.6)	2792 (9.7)	2935 (9.5)	28,826 (9.6)
45–54 yrs	12,831 (21.3)	6199 (21.4)	6526 (21.2)	64,166 (21.3)
55–64 yrs	16,054 (26.7)	7907 (27.4)	8046 (26.1)	80,017 (26.6)
65–74 yrs	13,928 (23.1)	6604 (22.8)	7203 (23.4)	69,691 (23.2)
75–84 yrs	8101 (13.5)	3768 (13.0)	4257 (13.8)	40,461 (13.5)
85 yrs +	3533 (5.9)	1633 (5.6)	1873 (6.1)	17,630 (5.9)
Marital status				
Married	30,766 (51.1)	14,769 (51.1)	15,744 (51.1)	152,775 (50.8)
Not married	29,451 (48.9)	14,134 (48.9)	15,096 (48.9)	148,016 (49.2)
Level of education				
Low	18,755 (31.1)	8844 (30.6)	9684 (31.4)	100,623 (33.5)
Middle	22,808 (37.9)	10,987 (38.0)	11,689 (37.9)	115,079 (38.3)
High	16,626 (27.6)	8143 (28.2)	8420 (27.3)	73,246 (24.4)
Missing	2028 (3.4)	929 (3.2)	1047 (3.4)	11,843 (3.9)
CCI				
0	50,825 (84.4)	24,447 (84.6)	25,985 (84.3)	255,689 (85.0)
1	4788 (8.0)	2249 (7.8)	2483 (8.1)	24,597 (8.2)
2	2879 (4.8)	1398 (4.8)	1470 (4.8)	13,350 (4.4)
3+	1725 (2.9)	809 (2.8)	902 (2.9)	7155 (2.4)
No. of previous IHD events				
0	57,877 (96.1)	27,775 (96.1)	29,641 (96.1)	288,263 (95.8)
1	1421 (2.4)	679 (2.3)	732 (2.4)	7598 (2.5)
2+	919 (1.5)	449 (1.6)	467 (1.5)	4930 (1.6)

Characteristics of the study population by all women with breast cancer (BC), women with right-sided BC, women with left-sided BC, and women without BC diagnosis

*No.* Number, *FU* follow-up, *yrs* years, *SD* standard deviation, *CCI* Charlson Comorbidity Index

Patient characteristics for all women with BC and by laterality are shown in Table 2. There were numerically more women with left-sided than right-sided BC, and the tumor characteristics were similar between these groups. A majority of the women with BC had a pathological tumor (T) stages 1 and 2 (46.7% and 30.2%, respectively). The pathological axillary lymph node stage was reported for 87.1% of the patients. Of those with reported data, 31.3% of the women were node-positive (N1–3+/N4+). Women with left-sided BC were less often operated with breast-conserving surgery (BCS), and there were also slightly fewer women with left-sided BC selected for RT compared to right-sided BC. No major differences regarding the use of endocrine treatment, chemotherapy, and trastuzumab were noticed between left-sided and right-sided BC.

**Table 2:** Patient characteristics for women with breast cancer by breast cancer laterality

BC characteristics	BC all, *n* (%)	BC right, *n* (%)	BC left, *n* (%)
No. of women	60,217 (100.0)	28,903 (100.0)	30,840 (100.0)
T status			
T0	8616 (14.3)	4224 (14.6)	4391 (14.2)
T1	28,100 (46.7)	13,527 (46.8)	14,289 (46.3)
T2	18,185 (30.2)	8646 (29.9)	9390 (30.4)
T3	2810 (4.7)	1329 (4.6)	1457 (4.7)
T4	1165 (1.9)	560 (1.9)	605 (2.0)
TX	1341 (2.2)	617 (2.1)	708 (2.3)
N status			
N0	33,659 (55.9)	16,226 (56.1)	17,155 (55.6)
N1–3	12,799 (21.3)	6154 (21.3)	6565 (21.3)
N4+	5993 (10.0)	2823 (9.8)	3120 (10.1)
NX	7766 (12.9)	3700 (12.8)	4000 (13.0)
ER status			
ER+	40,168 (66.7)	19,532 (67.6)	20,460 (66.3)
ER−	8666 (14.4)	4015 (13.9)	4587 (14.9)
Missing	11,383 (18.9)	5356 (18.5)	5793 (18.8)
PR status			
PR+	33,335 (55.4)	16,216 (56.1)	16,955 (55.0)
PR−	14,873 (24.7)	7033 (24.3)	7765 (25.2)
Missing	12,009 (19.9)	5654 (19.6)	6120 (19.8)
Surgery			
No surgery	2345 (3.9)	1115 (3.9)	1212 (3.9)
BCS	32,583 (54.1)	15,873 (54.9)	16,456 (53.4)
Mastectomy	23,917 (39.7)	11,268 (39.0)	12,453 (40.4)
Missing	1372 (2.3)	647 (2.2)	719 (2.3)
RT stratified for pathological node stage			
No RT	22,790 (37.8)	10,808 (37.4)	11,794 (38.2)
RT N0	21,524 (35.7)	10,472 (36.2)	10,887 (35.3)
RT N1–3	9088 (15.1)	4360 (15.1)	4658 (15.1)
RT N4+	4679 (7.8)	2219 (7.7)	2423 (7.9)
RT NX	2136 (3.5)	1044 (3.6)	1078 (3.5)
Endocrine therapy			
Yes	36,893 (61.3)	17,884 (61.9)	18,867 (61.2)
No	23,324 (38.7)	11,019 (38.1)	11,973 (38.8)
Chemotherapy			
Yes	16,552 (27.5)	7980 (27.6)	8528 (27.7)
No	43,665 (72.5)	20,923 (72.4)	22,312 (72.3)
Trastuzumab^a^			
Yes	1943 (7.5)	919 (7.3)	1024 (7.6)
No	23,964 (92.5)	11,595 (92.7)	12,368 (92.4)

Patient characteristics for women with breast cancer (BC) by BC laterality

*No.* number, *T* pathological tumor stage, *N* pathological lymph node stage, *ER* estrogen receptor, *PR* progesterone receptor, *BCS* breast-conserving surgery, *RT* radiotherapy

^a^Included women with BC registered from 2005 and later

### Risk of IHD for women with BC

**Fig. 1:**
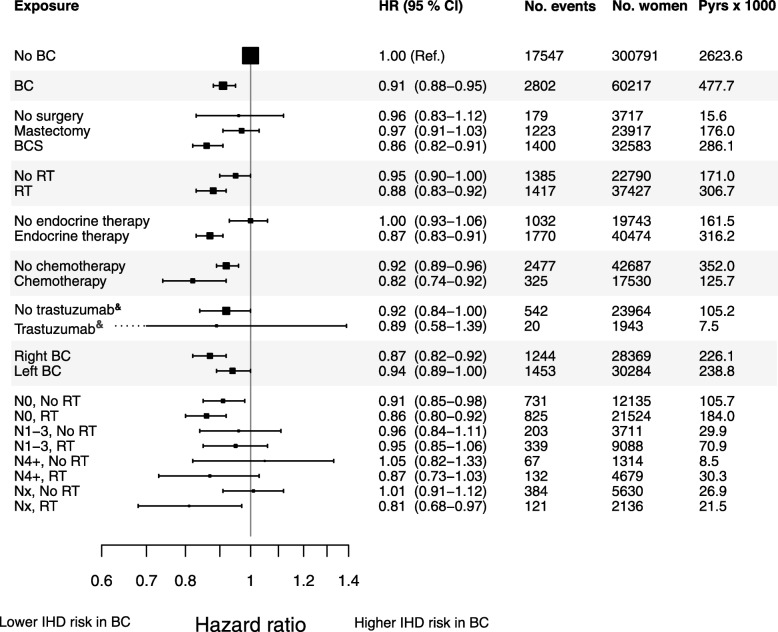
Risk of ischemic heart disease in women with breast cancer compared to women without breast cancer. The risk of ischemic heart disease (IHD) in women with breast cancer (BC) compared to women without BC diagnosis. Women with BC were stratified for the type of surgery, radiotherapy (RT), endocrine therapy, chemotherapy, treatment with trastuzumab, laterality of BC, and pathological lymph node stage. Adjusted for the number of previous IHD events, time since last IHD event, Charlson Comorbidity Index (IHD removed), and educational level. HR, hazard ratio; CI, confidence interval; No., number; Pyrs, patient years; Ref., reference; BCS, breast-conserving surgery. ^&^Included women registered from 2005 and later

The risk of IHD for women with BC compared to women without BC is presented in Fig. 1, along with the number of IHD events, the number of women, and the person-years at risk. The risk of IHD was lower for women with BC compared to women without BC with a HR of 0.91 (95% CI 0.88–0.95). Women with BC were stratified for different BC treatments and compared to women without BC. Women who received RT had a HR of 0.88 (95% CI 0.83–0.92), and women who received no RT of 0.95 (95% CI 0.90–1.00) for IHD compared to women without BC. Analysis concerning surgery, other adjuvant therapies, and stratifications by pathological lymph node stages are displayed in Fig. 1.

### Risk of IHD for women with BC by laterality

**Fig. 2:**
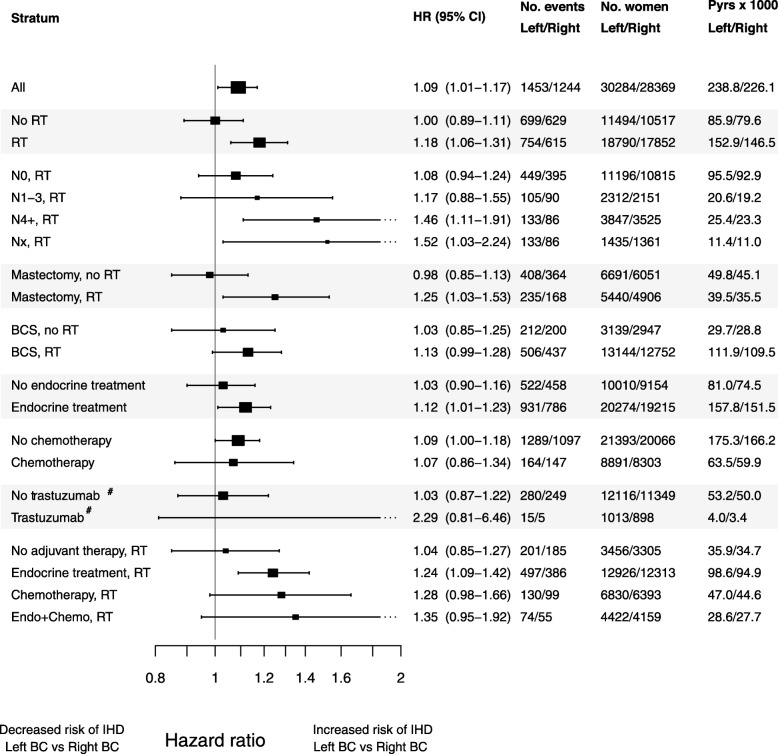
Risk of ischemic heart disease in women with left-sided breast cancer compared to right-sided. The risk of ischemic heart disease (IHD) in women with left-sided breast cancer (BC) compared to right-sided BC. The women were stratified for radiotherapy (RT), RT in relation to pathological lymph node status, type of surgery, endocrine therapy, chemotherapy, and treatment with trastuzumab. Adjusted for the number of previous IHD events, time since the last IHD event, Charlson Comorbidity Index (IHD removed), and educational level. HR, hazard ratio; CI, confidence interval; No., number; Pyrs, patient years; BCS, breast-conserving surgery. ^&^Included women registered from 2005 and later

The risk of IHD in women with left-sided BC compared to right-sided BC is shown in Fig. 2, along with the number of IHD events, the number of women, and the person-years at risk. There was a higher risk of IHD in left-sided BC compared to right-sided BC with a HR of 1.09 (95% CI 1.01–1.17). For women not receiving RT, the risk of IHD in left-sided and right-sided BC was very similar (HR 1.00, 95% CI 0.89–1.11) and without change when subdivided according to surgery. In women receiving RT, a higher risk of IHD in left-sided BC was seen, with a HR of 1.18 (95% CI 1.06–1.31). The left/right HR for IHD was 1.25 (95% CI 1.03–1.53) after mastectomy and RT and 1.13 (95% CI 0.99–1.28) after BCS and RT. Subgroup analyses according to pathological lymph node stage showed an increasing risk of IHD in left-sided BC compared to right-sided BC in more advanced lymph node stages, with the largest IHD risk seen in patients with four or more metastatic lymph nodes, HR 1.46 (95% CI 1.11–1.91). We found a higher left/right HR for IHD after RT in women receiving additional systemic adjuvant therapy; the HR was 1.24 (95% CI 1.09–1.42), 1.28 (95% CI 0.98–1.66), and 1.35 (95% CI 0.95–1.92) for endocrine therapy, chemotherapy, and endocrine therapy and chemotherapy combined, respectively, compared to RT alone.

### Cumulative incidence of IHD

**Fig. 3:**
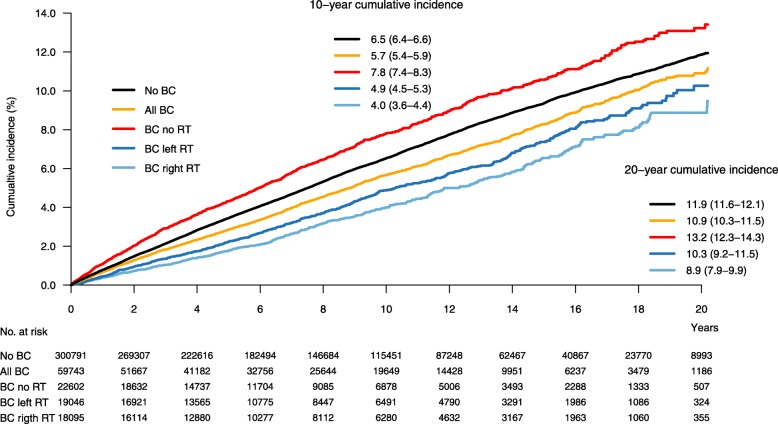
Cumulative incidence of ischemic heart disease. Cumulative incidence of ischemic heart disease (IHD) for women without breast cancer (BC) diagnosis, for all women with BC, for women with BC not receiving radiotherapy (RT), for women with BC receiving left-sided RT, and for women with BC receiving right-sided RT. No., number

The cumulative incidence of IHD during a 20-year follow-up for women without BC, all women with BC, women with BC not receiving RT, women receiving left-sided and right-sided RT is visualized in Fig. 3. The cumulative incidence of IHD was highest in women with BC who received no RT, followed by women without BC. In women who received RT, a higher cumulative incidence of IHD was seen in those receiving left-sided compared to right-sided RT. The difference between left- and right-sided RT was observed within the first years after RT, and it continued to increase with longer follow-up. The cumulative incidence rate of IHD at year 20 of follow-up was 11.9% for women without BC, 10.9% for all women with BC, 13.2% for women with BC not receiving RT, 10.3% for women receiving left-sided RT, and 8.9% for women receiving right-sided RT.

## Discussion

The present study showed no difference in comorbidity between women with BC and women without BC at baseline, but at follow-up, a significantly lower risk of IHD was seen in women with BC compared to women without BC. When women with BC receiving left-sided RT were compared to women receiving right-sided RT, a statistically significant 18% increased risk of IHD was seen. The HR’s for IHD in women with left-sided RT compared to right-sided RT were even higher in women with more extensive lymph node metastasis, and when endocrine therapy and chemotherapy were added to RT. In concordance with the findings in the Cox regression, the cumulative incidence of IHD was higher for women receiving left-sided RT compared to right-sided RT. The difference was observed within the first years after RT, and it continued to increase with longer follow-up.

In line with other studies [22, 23], a lower risk of IHD was seen in women with BC compared to women without BC. The analysis was adjusted for educational level, CCI, and the number of previous IHD events, but the findings may, however, be due to a confounding by a different distribution of lifestyle factors not captured by our adjustment. For example, some combinations of risk factors are negatively associated with IHD but positively with BC such as differences in child-bearing patterns, in the adoption of a healthy lifestyle and in adherence to mammography screening [24, 25].

The comparison to women without BC further shows that retrospective studies on cardiac toxicity will be biased if the background population is used as the control group.

The findings of lower risk of IHD in women with BC compared to women without BC were only observed in women recommended RT and other adjuvant therapies. Women with BC who received no RT had more comorbidities according to CCI, including previous IHD events, and a selection of healthier women to active therapies has probably occurred. Retrospective comparisons between women that did receive treatments with women that did not would likely be severely biased.

Thus, the risk of IHD after RT in BC could not be assessed by comparing BC patients with age-matched women without BC, nor by comparing women with BC who received RT to women with BC who did not. Instead, we chose to analyze the risk of IHD by comparing left- and right-sided BC. The mean heart doses and doses to coronary arteries are considerably higher after left-sided RT [6, 9, 26], but as cardiac structures also receive doses after right-sided RT, this comparison probably will underestimate the total added risk of IHD after RT in BC. In a recent systematic review of trials from 2010 to 2015, mean heart doses were 5.2 Gy and 3.7 Gy in left-sided and right-sided RT, respectively [11]. We recently showed considerably lower doses in a dosimetry study of 182 BC patients selected from the same registry as the present study, although including only patients undergoing coronary angiography due to a cardiac event [27]. The median mean heart dose was 0.6 Gy (interquartile range (IQR) 0.4–1.0) after right-sided RT and 2.7 Gy (IQR 1.7–4.2) after left-sided RT. Moreover, the left/right RT dose difference was higher in the mid and distal LAD, where median mean doses were 3.6 Gy (IQR 2.4–6.2) and 26.7 Gy (IQR 7.0–41.0) after left-sided RT, and 0.5 Gy (IQR 0.1–0.8) and 0.3 Gy (IQR 0.1–0.8) after right-sided RT, respectively [27].

A higher risk of IHD in women with BC receiving left-sided RT compared to right-sided RT has also been shown in previous studies [1, 4, 5, 8]. In this study, we show that this increase in risk persisted in women receiving RT for BC during 1992 to 2012. Most women were likely to receive three-dimensional conformal radiotherapy (3DCRT) given with tangential fields, but were treated before breathing adaption techniques, e.g., deep inspiratory breath-hold (DIBH), were implemented. The pattern of cumulative incidence further shows that the increase in the incidence of IHD in women with left-sided RT starts in the early years after RT and continues throughout follow-up.

An even higher risk of IHD was seen in women with more advanced pathological lymph node status. It is plausible that this group received more extensive RT including axillary and supraclavicular lymph nodes, and for some patients, the internal mammary chain (IMC), which is associated with higher cardiac radiation doses [6]. An increase in the risk of IHD was also seen when endocrine therapy and chemotherapy were added to RT in women with left-sided BC compared to right-sided BC. Anthracycline-based chemotherapy significantly increased the risk of cardiac death according to the 2005 EBCTCG meta-analysis, while tamoxifen use was associated with a lower cardiac death risk although the difference was not statistically significant (*p* = 0.06) [28]. A meta-analysis of published data from trials comparing aromatase inhibitors and tamoxifen reported a significant 30% increase in cardiovascular morbidity with aromatase inhibitors [29]. Whether aromatase inhibitors increase cardiovascular disease compared to no endocrine treatment is still unclear. Two recent reports [16, 17] indicate that adjuvant anthracycline treatment may increase the risk of IHD after RT in breast cancer, especially when combined with RT to the IMC [17]. Since women who received adjuvant endocrine therapy and chemotherapy also had more advanced pathological lymph node status, the increased risk of IHD in women receiving both RT and systemic therapy has to be interpreted with caution.

Strengths of this study include the population-based setting, the large size of the cohort, and the linkage with other registries enabling adjustments for comorbidity and socioeconomic factors.

Several limitations need mentioning. Causes of death were not analyzed since in women with BC, dying of BC is in this study design a competing risk to developing IHD, and the analysis comparing women with BC to women without BC diagnosis may bias the risk of IHD in women with BC. Another limitation is that information concerning individual radiation doses and targets were not available. However, most women received a dose of 2 to 50 Gy over 5 weeks, according to Regional BC treatment guidelines throughout most of the study period. A few patients may have received hypofractionated RT schedules of 2.66 to 42.56 Gy or of 2.67 to 40.05 Gy, as the guidelines allowed these schedules for selected cases from 2010. Most women with lymph node metastases also received RT to the axilla, the supraclavicular fossa, and in some cases the IMC, according to the Regional BC treatment guidelines. A further limitation is that the median follow-up of 8.1 years for women with BC may be too short to draw conclusions regarding the risk of IHD for those women receiving RT during the latter part of the inclusion period. Previous studies have shown that the risk of radiation-induced IHD begins within a few years from RT and continues for a long period of time, with the highest risk seen between 10 and 14 years from the RT [2], and it is thus likely that the elevation in risk of IHD in women receiving left-sided RT in the present study will be even more pronounced at longer follow-up. One cannot exclude the possibility that women with a history of left-sided BC are observed with more caution due to awareness of side effects of the RT, which may lead to more frequent cardiovascular examinations and thus an increased opportunity to detect IHD. On the other hand, there were proportionally slightly fewer women with left-sided BC selected for RT compared to right-sided BC in this study, suggesting an initial selection of healthier women for left-sided RT.

## Conclusions

We found an increase in the risk of IHD in women with left-sided BC compared to right-sided BC, given adjuvant RT during 1992 to 2012. Most of these women likely received 3DCRT given with tangential fields, but without DIBH techniques. The risk of radiation-induced IHD is expected to decrease when DIBH or other techniques that lower the cardiac and coronary doses are used. The results of this study emphasize the importance of fully implementing and further developing heart-sparing techniques, without compromising the target coverage and the beneficial effects of RT. Even though the rate of IHD events after RT is relatively low, long-term side effects of adjuvant treatment have to be taken into consideration, to ensure health and quality of life for BC survivors.

## Data Availability

Further information and data sets are available from the corresponding author on reasonable request.

## Abbreviations

RT: Radiotherapy
BC: Breast cancer
IHD: Ischemic heart disease
BCBaSe: Breast Cancer DataBase Sweden
AI: Aromatase inhibitors
NPR: National Patient Register
CCI: Charlson Comorbidity Index
ICD: International Classification of Disease
BCS: Breast-conserving surgery
HR: Hazard ratio
IMC: Internal mammary chain
